# KA101 outperforms other clinical adjuvants in inducing balanced Th1/Th2 immunity and robust B cell responses to varicella-zoster virus glycoprotein E

**DOI:** 10.3389/fimmu.2026.1849099

**Published:** 2026-07-16

**Authors:** Yaru Quan, Su Zhang, Wenwen Wang, Yiping Wang, Kangwei Xu, Kaiqin Wang, Wenyan Wan, Jiaojiao Nie

**Affiliations:** 1 Division of Respiratory Virus Vaccines, National Institutes for Food and Drug Control, Beijing, China; 2 The Center for Reference Material and Standardization, National Institutes for Food and Drug Control, Beijing, China; 3 State Key Laboratory of Drug Regulatory Science, National Institutes for Food and Drug Control, Beijing, China

**Keywords:** adjuvant, cellular immunity, innate immunity, multi-omics analysis, neutralizing antibody, varicella-zoster virus subunit vaccine

## Abstract

Reactivation of latent varicella-zoster virus (VZV) causes herpes zoster, which can further progress to persistent postherpetic neuralgia in a subset of patients. Rational adjuvant selection is critical for the development of recombinant VZV glycoprotein E (gE) subunit vaccines. In this study, we systematically evaluated the immunomodulatory properties of six adjuvants formulated with recombinant VZV gE in a mouse model. Multi-dimensional analyses, including innate and adaptive immunity, transcriptomics, and B-cell receptor (BCR) repertoire profiling, revealed that distinct adjuvants shape adaptive immune responses through divergent innate immune activation patterns. Traditional aluminum hydroxide and MF59 mainly induced Th2-biased humoral immunity but showed limited cellular immune activation. As TLR agonists, Poly(I:C) displayed a Th2-skewed profile with partial Th1 tendencies and exhibited oligoclonal B-cell expansion, whereas CpG 1018 potently promoted Th1 polarization and cytotoxic T-cell responses. Lipid nanoparticle (LNP) formulations drove chemokine-dependent inflammatory recruitment and facilitated dendritic cell maturation and humoral immune programming. Notably, KA101, a liposomal adjuvant containing MPL and QS-21, exhibited unique synergistic effects by inducing early IFN-γ production and simultaneously eliciting robust cellular immunity and high-titer neutralizing antibodies. Moreover, KA101 enhanced germinal center reactions, diversified the B-cell repertoire, and supported the establishment of long-term immune memory. Collectively, these findings provide mechanistic insights into adjuvant–antigen interactions and establish a rational framework for developing next-generation herpes zoster subunit vaccines with potent cellular and humoral immunogenicity and durable immune memory.

## 1 Introduction

Herpes zoster (HZ) results from the reactivation of latent varicella-zoster virus (VZV) that has established lifelong persistence in sensory ganglia following primary varicella infection. This reactivation is predominantly triggered by age-related decline in cell-mediated immunity and occurs with increased frequency in immunocompromised individuals, often accompanied postherpetic neuralgia (PHN) (1, 2). Accumulating evidence from large-scale cohort studies has unequivocally established that robust VZV-specific cell-mediated immunity constitutes the primary correlate of protection against viral reactivation and disease progression (3, 4).

Early vaccine development efforts focused on live-attenuated VZV formulations capable of inducing comprehensive cell-mediated immunity responses against multiple viral antigens (5, 6). This approach culminated in the licensure of Zostavax (ZVL; Merck Sharp & Dohme) (7), a landmark achievement that demonstrated 51% efficacy against HZ and 67% against PHN in adults ≥60 years of age (8, 9). However, its efficacy declined notably with increasing age at vaccination, from 70% in adults aged 50~59 years to 37% in those aged 70 years and above (10, 11), and protection waned substantially within 6~8 years post-vaccination (12, 13).

In contrast, the recombinant subunit vaccine Shingrix (GlaxoSmithKline), formulated with the abundant VZV glycoprotein E (gE) and the AS01B adjuvant system, elicits markedly enhanced immunogenicity and provides effective protection across all age groups, particularly in the elderly (14–16). The recent final analysis demonstrated that this protection lasts at least 11 years after two injections, with an overall vaccine efficacy of 79.8% in adults aged ≥50 years from approximately 6 to 11 years post-vaccination (17).The constituent simplicity of gE-based recombinant vaccines offers inherent advantages in terms of safety profile and manufacturing stability, as these non-replicating antigens eliminate risks associated with live-attenuated formulations. Nevertheless, protein antigens are inherently susceptible to enzymatic degradation and rapid clearance, necessitating the incorporation of immunopotentiating adjuvants to augment the magnitude, breadth, and durability of anti-gE immune responses (18).

Contemporary adjuvant development encompasses diverse molecular classes with distinct mechanisms of immunomodulation (16). Conventional aluminum hydroxide [Al(OH)_3_] primarily functions through antigen depot formation and NLRP3 inflammasome activation, predominantly eliciting humoral immunity with limited capacity for cellular immune induction (19, 20). The squalene-in-water emulsion MF59 enhances antigen uptake and presentation through robust recruitment of antigen-presenting cells (APCs) to the injection site and modulation of local cytokine milieu, thereby augmenting antibody titers while inducing a balanced Th1/Th2-polarized response (21, 22). Toll-like receptor (TLR) agonists represent a potent class of immunostimulants capable of activating dendritic cells (DCs) and amplifying adaptive immunity (23, 24). Specifically, CpG 1018, a TLR9 agonist, strongly drives Th1-type immunity and cytotoxic T lymphocyte (CTL) responses, as well as promotes the massive production of type I interferons (25). Poly(I:C), a dual agonist of TLR3 and MDA5, effectively mimics viral double-stranded RNA, potently activating DCs and enhancing cross-presentation capabilities (26, 27). Lipid nanoparticles (LNPs), originally developed as nucleic acid delivery vehicles (28), exhibit intrinsic adjuvanticity through ionizable lipid components that activate innate immunity and induce inflammatory cytokines, potentially involving TLR and inflammasome signaling pathways (29, 30). Composite adjuvant systems represented by AS01 [such as KA101 adopted in this study, a liposomal formulation containing monophosphoryl lipid A (MPL) and QS-21] synergistically activate TLR4 signaling and the NLRP3 inflammasome, inducing early IFN-γ production and efficiently priming antigen-specific CD4^+^ T cells with Th1 polarization alongside robust CD8^+^ T cell responses. Their ability to induce robust and long-lasting humoral and cellular immunity has been confirmed in approved VZV vaccines, respiratory syncytial virus vaccines, and malaria vaccines (31–34).

Despite the diverse immunomodulatory mechanisms represented by these adjuvants, systematic head-to-head comparisons evaluating their effects on the same antigen within a unified experimental framework remain scarce. Such comparative studies are essential to elucidate how distinct innate activation pathways shape the quality and durability of adaptive immune responses. In this study, we formulated recombinant VZV gE protein with six mechanistically distinct adjuvants—Al(OH)_3_, MF59, CpG 1018, Poly(I:C), LNP, and KA101—and performed integrative analyses of innate immunity, humoral immunity, cellular immunity, transcriptomics, and BCR repertoire profiling in a murine model. Our primary goal was to characterize adjuvant-specific innate immune activation patterns and delineate their downstream effects on Th1/Th2 polarization, germinal center dynamics, and long-term memory formation. Together, these findings are expected to provide a mechanistic basis for rational adjuvant selection in VZV subunit vaccines development.

## 2 Materials and methods

### 2.1 Antigens and adjuvants

Recombinant VZV gE antigen and adjuvants including aluminum hydroxide, MF59, CpG 1018, LNP, and KA101 were supplied by Sinovac, whereas Poly(I:C) was purchased from Kaiping Genuine Biochemical Pharmaceutical Co., Ltd. (Kaiping, China). Antigen-adjuvant formulations were prepared by mixing equal volumes (1:1, v/v).

### 2.2 Animal immunization

Female C57BL/6J mice (6~8 weeks old) were randomly allocated into 14 groups (n = 8 per group): PBS control, gE alone, six adjuvant-only, and six gE-adjuvant combinations. All animals were immunized intramuscularly on days 0 and 14 with 100 μL vaccine containing 2.0 μg gE protein. Blood was collected at 20 h post-prime for innate immunity analysis, and again on days 14 and 28 for humoral response evaluation. On day 28, mice were euthanized via gradual CO_2_ displacement at a rate of 20%~30% chamber volume per minute, following AVMA euthanasia guidelines. Spleens were harvested immediately after the confirmation of death for cellular immunity, transcriptome sequencing, and antibody repertoire studies. Animal research protocols were approved by the Institutional Animal Care and Use Committee at the National Institutes for Food and Drug Control, China (Approval number: 2024[B]051).

### 2.3 Assessment of innate immune activation

Serum samples obtained at 20 hours post-primary immunization were analyzed by Luminex multiplex assay (Invitrogen, USA) for concentrations of IFN-α, IFN-γ, TNF-α, IL-6, IL-1β, IL-12p70, MCP-1, IP-10, MIP-1α, and IL-10, to evaluate innate immune activation and inflammatory responses.

### 2.4 Evaluation of humoral immune responses

#### 2.4.1 Determination of binding antibody titers

Serum levels of gE-specific binding antibodies were measured by indirect enzyme-linked immunosorbent assay (ELISA). Microtiter plates were coated with gE antigen (5 μg/mL, 100 μL/well) overnight at 2~8 °C. After blocking and washing, serum samples were serially diluted and incubated at 37 °C for 90 min, followed by HRP-conjugated secondary antibody (1:20,000; Seracare, USA) incubation for 60 min. Substrate solution (Qinbang, China) was added and incubated in the dark for 15 min. The reaction was terminated with stop solution, and absorbance was measured at 450 nm and 630 nm (OD_450-630_). Samples were considered positive when OD values were ≥ 2.1-fold the mean OD of negative controls. Antibody titers were expressed as geometric mean titers (GMT).

#### 2.4.2 Determination of neutralizing antibody titers

VZV-neutralizing antibody titers were determined using a cytopathic effect (CPE)-based microneutralization assay. Serum specimens were heat-inactivated at 56 °C for 30 min and then serially diluted two-fold in MEM supplemented with 1% penicillin-streptomycin. Each dilution was mixed with an equal volume of VZV suspension containing 100 CCID_50_ and incubated at 37 °C with 5% CO_2_ for 2 h. Thereafter, 100 μL of SV-1 cell suspension (1 × 10^5^ cells/mL) was added to each well, and the plates were maintained at 37 °C with 5% CO_2_ for 7 days, with daily examination for CPE. The neutralizing antibody titer was expressed as the reciprocal of the highest serum dilution that completely prevented CPE. In instances where partial CPE was observed at consecutive dilutions, the geometric mean titer was calculated and adopted as the final value.

#### 2.4.3 Determination of gE-specific IgG subclasses

gE-specific IgG subclass concentrations in mouse serum were determined by indirect ELISA. Microtiter plates were coated with 0.1 μg/well of gE antigen or serial dilutions of IgG subclass standards (concentration range: 1000~0.49 pg/μL) in PBS and incubated overnight at 4 °C. The IgG2c Fc standard was purchased from Abcam (UK), while all other IgG subclass standards were obtained from Sino Biological (China). After discarding the coating solution, plates were washed three times with PBST and blocked with 300 μL/well of 5% (w/v) skim milk in PBS at room temperature for 1~2 h. Following washing, serum samples diluted in 1% skim milk/PBST were added (100 μL/well) and incubated at room temperature for 2 h. Plates were then washed five times with PBST, and HRP-conjugated IgG subclass-specific secondary antibodies (Proteintech, China) diluted 1:5000 in PBST were added (100 μL/well) and incubated at room temperature for 1 h. After washing, substrate solution was added and incubated in the dark at room temperature for 15 min. The reaction was terminated by adding stop solution, and absorbance was measured at 450 nm and 630 nm (OD_450_–OD_630_). Concentrations of each IgG subclass were calculated from the corresponding standard curves.

### 2.5 Analysis of T cell subsets

Flow cytometry was employed to evaluate antigen-specific T cell responses in mouse spleens, with all reagents purchased from Biolegend (USA). Spleens were aseptically harvested on day 14 after the second immunization, mechanically disrupted, filtered through a 40 μm cell strainer, and centrifuged at 350 ×g for 10 min. Red blood cells were lysed with RBC lysis buffer on ice for 4~5 min, and the remaining cells were washed with PBS containing 2% FBS and resuspended in RPMI-1640 complete medium. Splenocytes were stimulated with 10 μg/mL gE peptide pool in the presence of 0.1% brefeldin A (BFA) at 37 °C with 5% CO_2_ for 12 h. Following stimulation, cells were stained with near-infrared fixable viability dye to exclude dead cells. After Fc receptor blocking with anti-CD16/CD32 antibody for 10 min, surface markers (CD3, CD4, CD8, and CD40L) were stained at 4 °C in the dark for 20 min. For intracellular cytokine staining, cells were fixed, permeabilized, and stained with antibodies against IFN-γ, TNF-α, IL-2, and IL-4 at room temperature in the dark for 20 min. Samples were acquired on a flow cytometer, and the frequencies of cytokine-producing T cell subsets were analyzed.

### 2.6 Transcriptome sequencing and bioinformatic analysis

For each experimental group, spleen samples from eight mice were collected and pooled in pairs to generate four biological replicates, three of which were selected for RNA sequencing. Raw reads were filtered and mapped to the mouse reference genome (GRCm39) using HISAT2. Gene expression levels were quantified as transcripts per million (TPM) using StringTie. Differentially expressed genes (DEGs) were identified using a stratified screening strategy. For group comparisons with abundant transcriptional differences, a stringent threshold of |log_2_FC| ≥ 0.585 and FDR ≤ 0.05 was applied. For comparisons with limited DEGs under the stringent criterion, an exploratory threshold of |log_2_FC| ≥ 0.585 and raw P ≤ 0.05 was adopted to capture additional candidate genes for downstream pathway enrichment analysis. All identified DEGs were subjected to Gene Ontology (GO) enrichment analysis and Kyoto Encyclopedia of Genes and Genomes (KEGG) pathway analysis to elucidate the biological processes and signaling pathways involved. To focus on functionally relevant immune responses, immune-related pathways were extracted using immune-specific keywords (e.g., cytokine, interferon, TLR, antigen presentation, T cell, B cell) and immune gene set annotations. Enriched pathways were sorted by ascending P value to prioritize the most statistically significant immune signaling pathways. To visualize expression profiles of immune-related genes across adjuvant formulations, hierarchically clustered heatmaps were generated. Genes were selected based on their annotation to the immune-related pathways identified above. TPM expression values of these genes were normalized by row-wise Z-score and visualized using Hiplot (https://hiplot.com.cn). Unsupervised hierarchical clustering of genes was implemented with Euclidean distance and Ward.D2 linkage.

### 2.7 Antibody repertoire analysis

Similarly, spleen samples from eight mice per group were pooled in pairs to obtain four biological replicates. Three replicates were used for total RNA extraction and subsequent cDNA synthesis. B cell receptor (BCR) heavy chain complementarity-determining region 3 (CDR3) regions were subjected to high-throughput immunorepertoire sequencing. Processed sequences were aligned to reference gene sequences using MiXCR following quality control, and VDJtools was used to analyze CDR3 length distribution, V gene usage, V-J pairing patterns, clonal diversity, and expansion levels.

### 2.8 Statistical analysis

Statistical analyses were conducted using GraphPad Prism version 8.0 (GraphPad Software, USA). Continuous data were expressed as mean ± standard deviation (SD), whereas antibody titers were reported as geometric mean with 95% confidence interval (95% CI). To meet the assumption of normality, antibody titers were log-transformed prior to analysis. Comparisons among multiple groups were performed by one-way analysis of variance (ANOVA) with Tukey’s *post-hoc* test for multiple comparisons. Statistical significance was denoted as *p < 0.05, **p < 0.01, ***p < 0.001, and ****p < 0.0001.

## 3 Results

### 3.1 Adjuvants elicit distinct innate immune activation profiles

**Figure 1:**
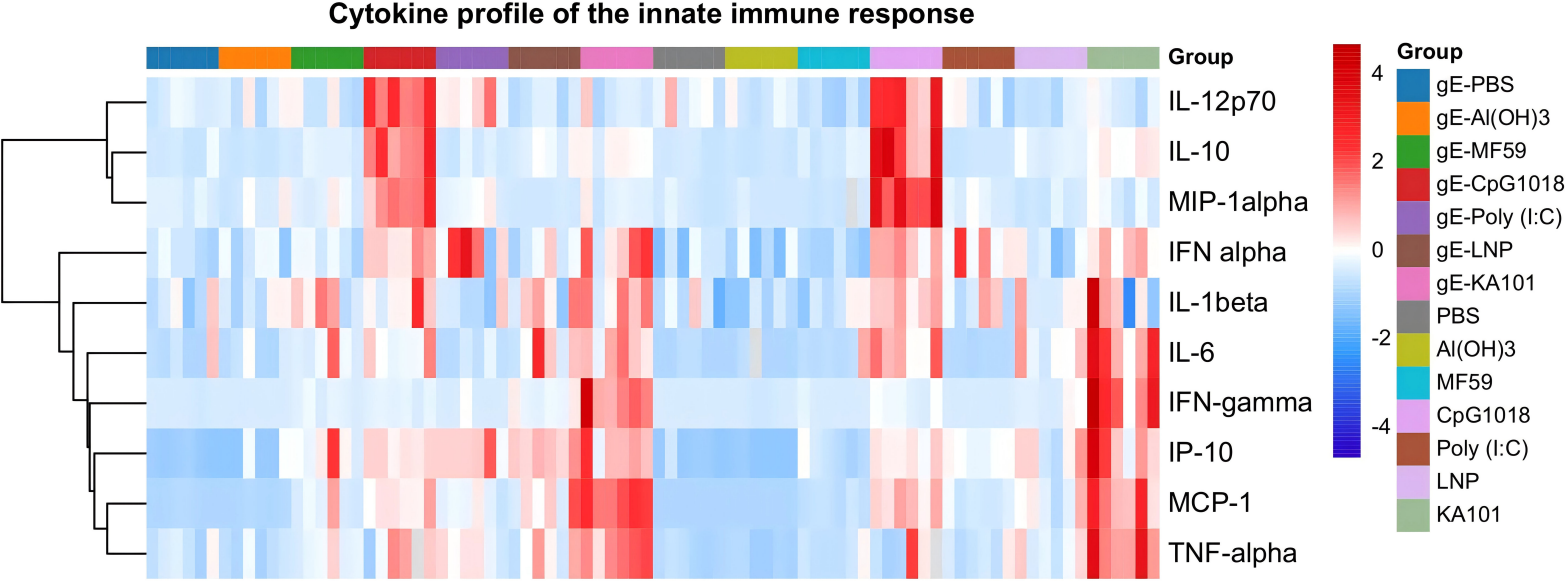
Cytokine and chemokine profiles of innate immunity induced by various adjuvants and formulations. The heatmap depicts the relative expression levels of each analyte across all experimental groups. Data were row-scaled (Z-score normalization), and hierarchical clustering (Ward.D2 method with Euclidean distance) was performed to group adjuvants with similar cytokine induction profiles. The color scale represents relative levels from low (blue) to high (red). gE-XXX: Groups immunized with gE antigen formulated with the indicated adjuvant. XXX: Groups administered with the adjuvant alone (without antigen).

To systematically compare the innate immune activation signatures induced by different adjuvants, serum samples were collected from mice at 20 h post-primary immunization. Key inflammatory cytokines and chemokines were quantified using Luminex technology (**Supplementary Figure 1**), and the data were visualized by hierarchical clustering heatmap following normalization, Ward’s minimum variance method, and Euclidean distance analysis (**Figure 1**). All adjuvant-containing groups significantly upregulated multiple cytokines compared to the PBS control group, confirming effective innate immune activation.

Among conventional adjuvants, aluminum hydroxide [Al(OH)_3_] elicited relatively modest responses, mechanistically consistent with its limited capacity to induce cellular immunity. MF59 triggered a broad yet mild inflammatory profile characterized by elevated IL-6, MCP-1, IL-1β, and IP-10, suggesting that its adjuvant effect may be mediated through local tissue stress and subsequent cellular recruitment. TLR agonists displayed pathway-specific signatures. CpG 1018, a TLR9 agonist, demonstrated potent Th1-polarizing potential, inducing high levels of IFN-α accompanied by robust upregulation of IL-12p70, IP-10, and TNF-α. IL-12p70 serves as a critical instructive signal driving naïve T cell differentiation toward the Th1 lineage, while IP-10 recruits CXCR3^+^ Th1 and cytotoxic T cells, collectively establishing a microenvironment conducive to cellular immunity. Poly(I:C), a TLR3 agonist, similarly mimics viral nucleic acids and induced a response profile resembling that of CpG 1018, albeit with significantly lower magnitude. This differential intensity likely reflects distinct expression patterns of TLR3 versus TLR9 among APC subsets, or divergent downstream signal transduction efficiencies.

Novel delivery systems and immunomodulators exhibited unique activation patterns. LNP formulations displayed a chemokine-driven response characterized by prominent MCP-1 upregulation, accompanied by broad elevations in TNF-α, IL-6, and IL-1β, suggesting that their primary mechanism involves efficient recruitment and activation of monocytes and macrophages. KA101 concurrently induced high levels of MCP-1, IP-10, IFN-γ, and IFN-α. Its capacity for early IFN-γ induction suggests potential for rapid activation of unconventional immune cells, such as innate lymphoid cells or memory-phenotype T cells, implying that its mechanism may involve integration of multiple intracellular signaling pathways, thereby inducing a more comprehensive and Th1-biased immune response.

Collectively, these findings demonstrate that different adjuvants activate divergent innate immune signaling pathways, generating characteristic cytokine milieus that likely shape the subsequent polarization and magnitude of adaptive immune responses.

### 3.2 Adjuvants shape the magnitude and quality of humoral immune responses

**Figure 2:**
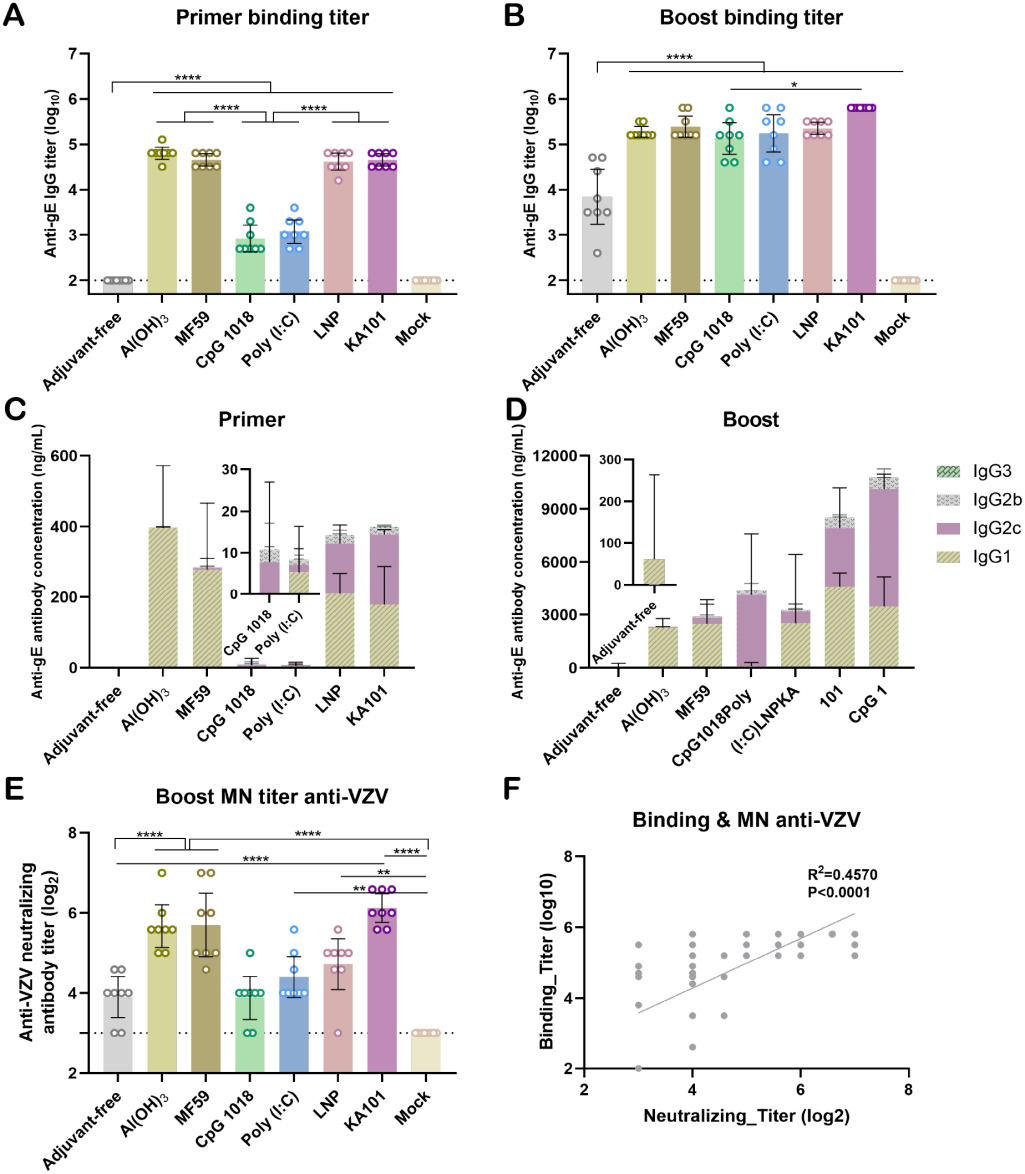
Adjuvants modulate the magnitude, subclass profile, and neutralizing activity of gE-specific antibody responses. **(A, B)** Serum anti-gE IgG titers were measured by ELISA 14 days after prime **(A)** and boost **(B)** immunization. Data are presented as geometric mean titers (GMT) with 95% confidence intervals (n = 8 mice per group). **(C, D)** Concentrations of gE-specific IgG subclasses (IgG1, IgG2c, IgG2b, and IgG3) after prime **(C)** and boost **(D)** immunization. The insets show magnified views of the low−titer groups to clearly visualize baseline antibody levels. **(E)** Neutralizing antibody titers against VZV in post-boost sera, as determined by microneutralization assay. Titers are expressed as log_2_ values. **(F)** Correlation between gE-specific binding antibody titers (log_10_) and neutralizing antibody titers (log_2_) against VZV. Each symbol represents an individual mouse. The p-value and coefficient of determination (R²) are shown.

To systematically evaluate adjuvant-mediated modulation of humoral immunity, gE-specific antibody responses were assessed at 14 days post-primary immunization (prime) and 14 days post-boost (boost). All adjuvant formulations significantly enhanced gE-specific IgG titers compared to antigen alone at both time points (**Figures 2A, B**). Notably, CpG 1018 and Poly(I:C) induced lower antibody titers following priming (p < 0.0001), yet achieved comparable levels post-boost, indicating delayed antibody kinetics without compromising ultimate response magnitude.

To delineate adjuvant effects on immune polarization, IgG subclass distributions (IgG1, IgG2b, IgG2c, and IgG3) were analyzed (**Figures 2C, D**). Marked heterogeneity in Th1/Th2 bias was observed across formulations. Aluminum hydroxide (Al[OH]_3_) elicited a pronounced Th2-skewed response, with IgG1 as the dominant subclass. Conversely, CpG 1018 induced robust Th1-polarized immunity, characterized by elevated IgG2c proportions. MF59 and Poly(I:C) exhibited a Th2-skewed profile, although its IgG2c levels were notably higher than those induced by Al(OH)_3_. KA101 and LNP generated comparatively balanced Th1/Th2 profiles, with KA101 displaying the highest overall antigen-specific antibody titers among all adjuvants and substantial levels of both IgG2c and IgG1.

Neutralizing antibody titers against VZV were assessed using a microneutralization assay (**Figure 2E**). Notably, the Al(OH)_3_, MF59, and KA101 groups induced the most robust neutralizing antibody responses. Consistent with the binding antibody data, a strong positive correlation was observed between gE-specific binding antibody titers and VZV neutralizing antibody titers across all groups (p < 0.0001; **Figure 2F**). Interestingly, despite their distinct polarization profiles—CpG 1018 inducing strong Th1 bias, and Poly(I:C) and LNP eliciting balanced or Th2-skewed responses—these three adjuvant groups induced only moderate levels of neutralizing antibodies. This suggests that while these adjuvants may effectively enhance cellular immunity, their capacity to promote neutralizing antibody responses is relatively limited. In contrast, KA101 uniquely combined strong Th1 polarization with high neutralizing antibody titers, demonstrating its potential to coordinately elicit both cellular and humoral immune responses.

### 3.3 Adjuvants drive distinct antigen-specific T cell responses

**Figure 3:**
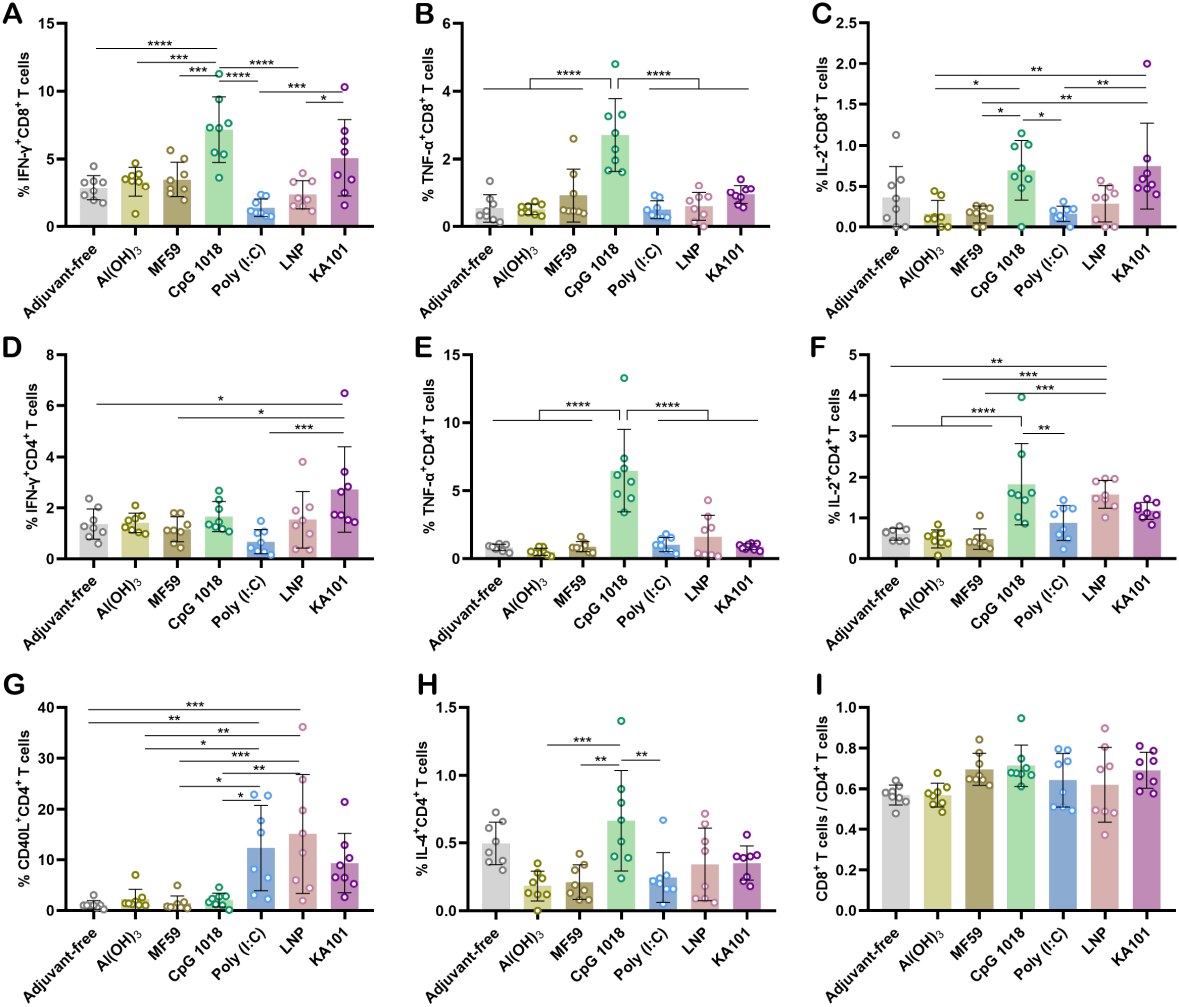
Adjuvants drive divergent antigen-specific T cell responses. Flow cytometric analysis of splenocytes from immunized mice. **(A–C)** Frequencies of cytokine-producing antigen-specific CD8^+^ T cells (gated on CD3^+^CD8^+^ T cells). **(D–H)** Frequencies of cytokine-producing antigen-specific CD4^+^ T cells (gated on CD3^+^CD4^+^ T cells). **(I)** Ratio of CD8^+^ to CD4^+^ T cells among CD3^+^ T cells. Mice were immunized with antigen alone or antigen formulated with the indicated adjuvants.

To evaluate the modulatory effects of different adjuvants on cellular immunity, antigen-specific T cell responses in the spleen were systematically analyzed by flow cytometry (**Supplementary Figure 2**). Marked differences in both the magnitude and functional quality of T cell responses were observed across adjuvant groups (**Figure 3**). CpG 1018 exhibited a superior capacity to elicit cellular immunity, characterized by the highest frequencies of IFN-γ^+^, TNF-α^+^, and IL-2^+^ cells among antigen-specific CD8^+^ T cells, as well as the highest proportion of CD8^+^ T cells within the total T cell population. However, this formulation failed to induce appreciable CD40L expression on CD4^+^ T cells, consistent with its limited capacity to support humoral immunity as evidenced by diminished neutralizing antibody titers. In contrast, KA101 induced a more comprehensive cellular immune profile, simultaneously generating high frequencies of IFN-γ^+^, IL-2^+^, and IL-4^+^ cells among both CD4^+^ and CD8^+^ T cell subsets. Notably, this was accompanied by a trend toward increased CD40L^+^CD4^+^ T cell responses, suggesting its potential to coordinately activate both cellular and humoral immunity. LNP significantly enhanced antigen-specific IL-2^+^CD4^+^ and CD40L^+^CD4^+^ T cell responses, while Poly(I:C) also elicited robust CD40L^+^CD4^+^ T cell responses. Among conventional adjuvants, Al(OH)_3_ and MF59 induced levels of cellular immunity comparable to the antigen-alone group, further reinforcing their characterization as Th2-biased humoral adjuvants with limited capacity to promote cellular responses. Collectively, these findings demonstrate that adjuvant selection critically determines the magnitude, functional quality, and lineage polarization of antigen-specific T cell responses.

### 3.4 Transcriptomic profiling of spleen reveals adjuvant-specific immune signatures

**Figure 4:**
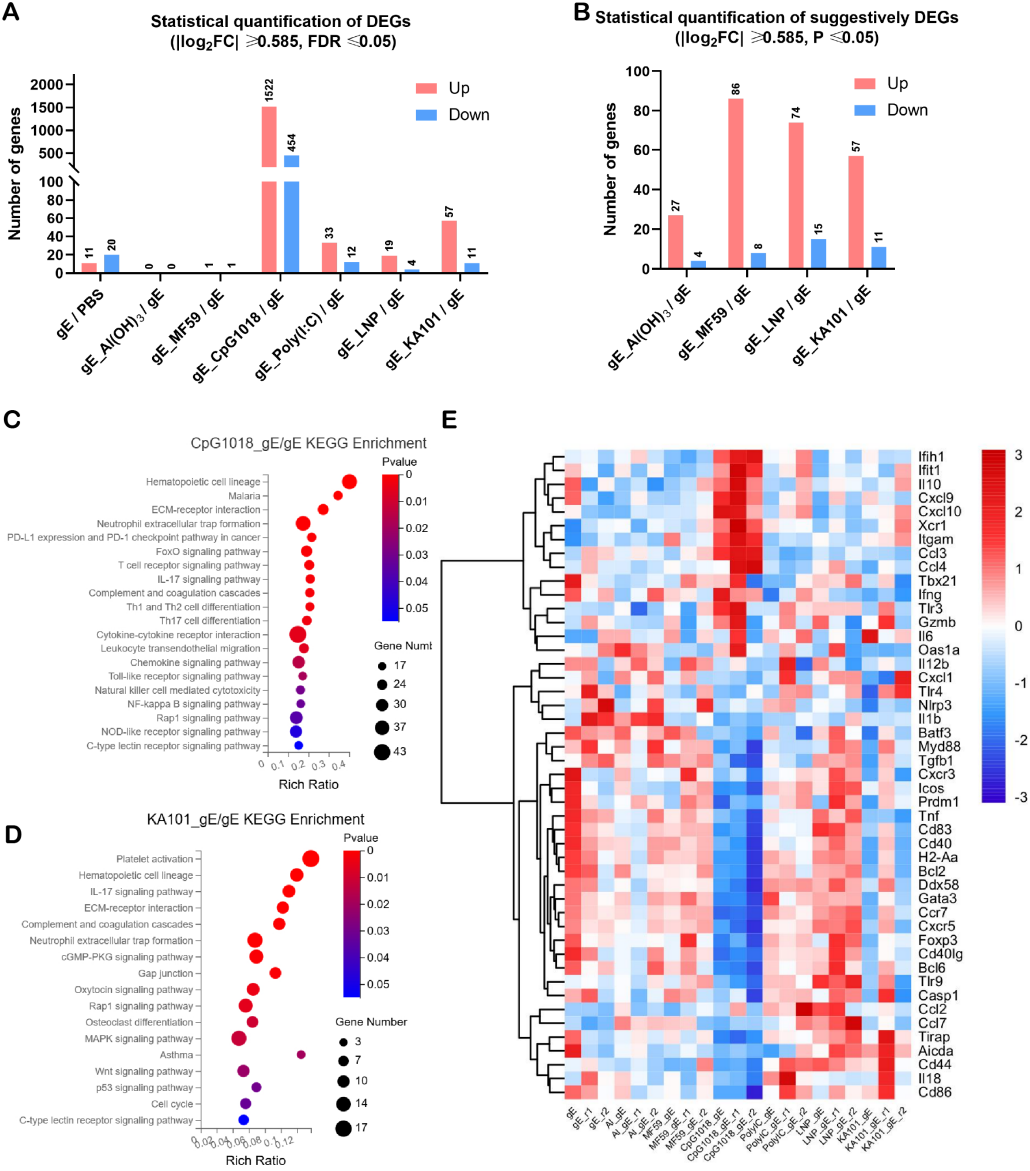
Transcriptomic profiling and functional enrichment analysis in mice immunized with adjuvanted gE vaccines. Mice were immunized twice with gE protein alone or gE formulated with different adjuvants. Splenic transcriptomes were analyzed two weeks after the booster immunization. **(A, B)** Quantitative analysis of differentially expressed genes (DEGs). Panel a shows the number of stringent DEGs (|log_2_FC| ≥ 0.585, FDR Q ≤ 0.05), while panel b shows the number of suggestively differentially expressed genes (|log_2_FC| ≥ 0.585, nominal P ≤ 0.05). In both panels, bars are colored to represent up-regulated (red) and down-regulated (blue) genes. **(C)** KEGG pathways for CpG 1018_gE vs. gE. **(D)** KEGG pathways for KA101_gE vs. gE. **(E)** Hierarchical clustering heatmap of immune-related gene expression in mouse splenic tissues. Expression levels of selected immune-related genes, derived from RNA-seq TPM values, are displayed after row-wise Z−score normalization. The color scale represents relative expression levels, ranging from low (blue) to high (red). Biological replicates for each treatment group are indicated as “_r1” and “_r2”. Gene symbols are shown on the right.

To characterize adjuvant-induced transcriptional programs, RNA sequencing was performed on spleens from gE-immunized mice. DEGs were identified using stringent (|log_2_FC| ≥ 0.585, FDR ≤ 0.05) and exploratory (|log_2_FC| ≥ 0.585, P ≤ 0.05) thresholds (**Figures 4A, B**). For CpG 1018 and Poly(I:C) groups with robust transcriptional changes, the stringent FDR threshold was applied. For the other groups (Al(OH)_3_, MF59, LNP, and KA101), where DEG numbers under the stringent criterion were limited, the exploratory P-value threshold was adopted to ensure adequate gene coverage for meaningful pathway enrichment.

The gE alone group showed limited transcriptional changes (11 upregulated genes) enriched in MAPK signaling pathway, Antigen processing and presentation, IL-17 signaling pathway, and TNF signaling pathway, indicating weak immunogenicity requiring adjuvant synergy. Al(OH)_3_ enriched C-type lectin receptor signaling pathway and antigen processing and presentation, with hierarchical clustering revealing concomitant activation of NLRP3 inflammasome components (*Casp1, Pycard*) and inflammatory chemokines (*Il1b, Il6, Cxcl1, Ccl7*) that collectively establish a microenvironmental basis for Th2-biased humoral immunity (**Figure 4E**). MF59 upregulated ECM-receptor interaction and Complement and coagulation cascades while downregulating Toll-like receptor signaling pathway and Antigen processing and presentation. Clustering analysis further showed that MF59 promoted dendritic cell maturation and migration through *Ccr7* and *H2-Aa*, drove multilineage T cell differentiation (*Tbx21, Gata3, Foxp3*), and enhanced germinal center formation (*Bcl6*), indicating Th2-biased humoral immunity with local microenvironment remodeling. CpG 1018 extensively enriched T cell receptor signaling pathway, Cytokine-cytokine receptor interaction, and NF-κB signaling pathway, driving lymphocyte proliferation and Th1 polarization. Clustering analysis corroborated this by revealing strong viral pattern recognition signatures (*Ifih1*) and type I interferon responses (*Ifit1, Oas1a*), driving Th1 polarization (*Tbx21, Ifng*) and cytotoxic effector function (*Gzmb*), accompanied by key chemokine expression (*Cxcl9, Cxcl10*). Poly(I:C) downregulated multiple immune activation pathways while upregulating antiviral and negative regulatory processes. Clustering analysis confirmed RIG-I activation (*Ddx58*) with concurrent suppression of inflammasome components (*Nlrp3, Il1b*) and cross-presentation factor Batf3, while driving pro-survival and memory signals (*Bcl2, Cd44*), suggesting a finely regulated adaptive phenotype. LNP enriched Natural killer cell mediated cytotoxicity and Fc gamma R-mediated phagocytosis pathways with concurrent downregulation of lymphocyte activation signals. Clustering analysis revealed multi-pathway synergistic activation engaging both TLR/MyD88 and RIG-I (*Ddx58*) signaling, with upregulation of costimulatory molecules (*Cd40/Cd40lg, Cd86, Icos*) and homing receptors (*Ccr7, Cxcr5*), preferentially driving a complete humoral immune program encompassing germinal center reactions (*Bcl6, Aicda*), plasma cell differentiation (*Prdm1*), and memory cell establishment (*Bcl2, Cd44*). KA101 enriched Hematopoietic cell lineage, IL-17 signaling pathway, Neutrophil extracellular trap formation pathways, promoting inflammatory responses and innate immune activation. Clustering analysis further demonstrated TLR4 adaptor (*Tirap*) engagement with robust type I interferon responses (*Cxcl9, Cxcl10*) and inflammatory signals (*Il18, Il6*), favoring Th1 polarization and cytotoxic potential while promoting germinal center reactions (*Aicda*) and long-term immune memory formation (*Cd44*).

Collectively, to visualize the expression patterns of immune-related genes across all adjuvant groups, we selected genes annotated to the immune pathways identified above and generated a hierarchical clustering heatmap (**Figure 4E**). These transcriptomic analyses revealed the molecular mechanisms by which distinct adjuvants orchestrate diverse immune response patterns, ranging from Th2-biased humoral immunity to Th1-polarized cellular responses, and encompassing germinal center promotion, inflammatory modulation, and integrated immune programming—providing a rational foundation for adjuvant selection in vaccine design. Detailed KEGG pathway and GO enrichment analyses are provided in **Figures 4C, D**; **Supplementary Tables 1**–**S5**, and **Supplementary Figure 3**.

### 3.5 Adjuvant-specific remodeling of the splenic B-cell receptor repertoire

**Figure 5:**
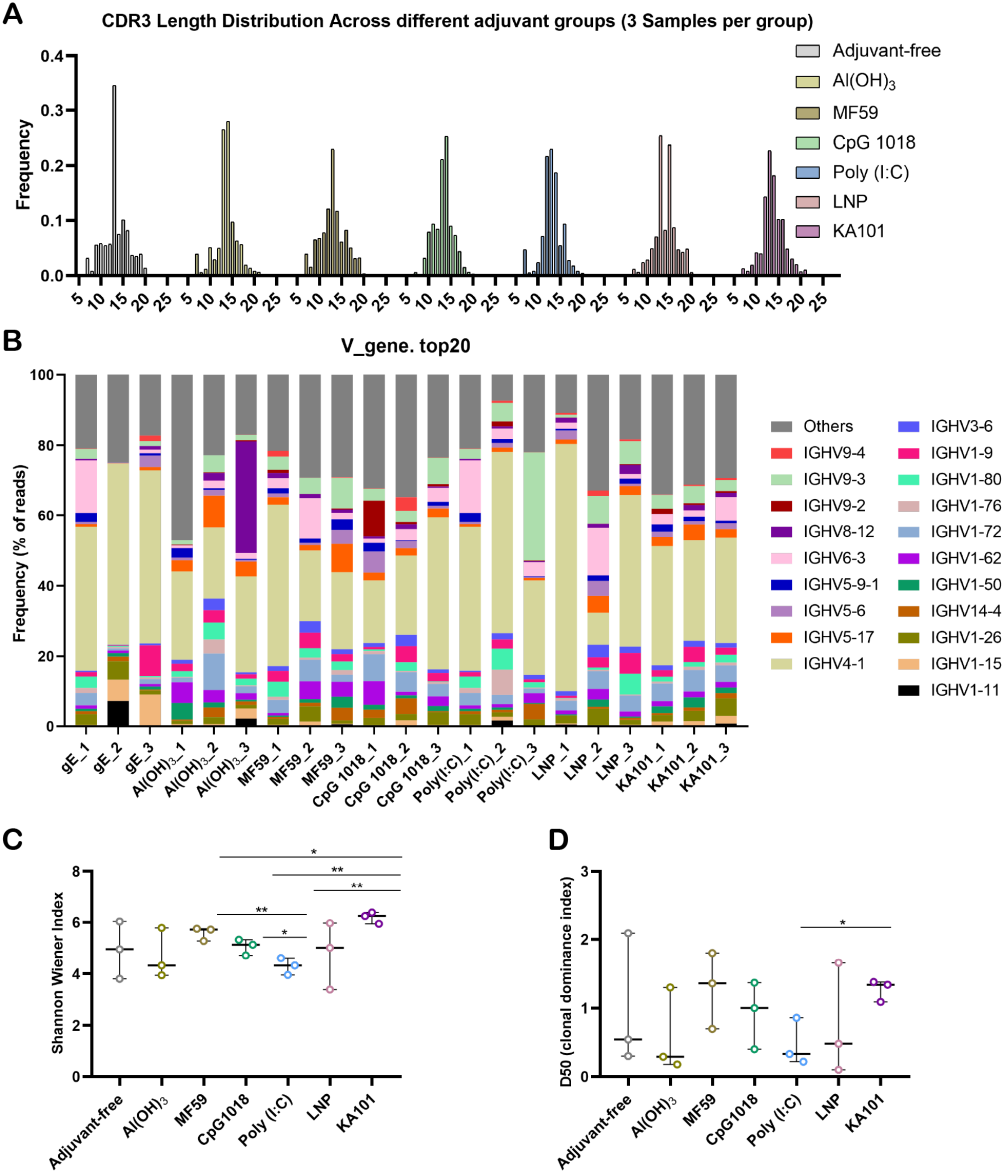
Adjuvant-dependent modulation of the splenic BCR immune repertoire. **(A)** CDR3 amino acid length distribution. **(B)** Frequency of the top 20 V gene families. **(C)** Shannon diversity index, quantifying clonal evenness and richness. **(D)** D50 index, defined as the minimal number of clones comprising 50% of the total repertoire, reflecting clonal dominance.

Total RNA was extracted from mouse spleens at 14 days post-secondary immunization for high-throughput sequencing of the BCR heavy chain CDR3. Accounting for pronounced inter-individual variability within the same group, nonparametric statistical approaches were applied for cross-group comparative analyses (**Figure 5**). CDR3 length distributions followed a Gaussian pattern across all samples, with peak lengths concentrated between 10 and 16 amino acids. Notably, the Poly(I:C) group exhibited a narrower and more focused distribution, suggestive of clonal expansion, whereas the MF59 group displayed a broader and more dispersed distribution, indicative of greater diversity. Remaining adjuvant formulations displayed intermediate distributional phenotypes. V gene segment utilization analysis demonstrated that the adjuvant-free control group predominantly utilized IGHV4-1 as the dominant family. In the aluminum adjuvant group, frequencies of IGHV1-62 and IGHV5-17 were increased. The MF59 group showed elevated usage of IGHV1-62, IGHV14-4, and IGHV9-3. In the KA101 group, reduced IGHV4-1 usage was accompanied by compensatory increases in IGHV1-72 and IGHV5-17. V-J junctional pairing analysis revealed that the control group featured predominant combinations of IGHV4-1/IGHJ3. The aluminum adjuvant group showed an increased frequency of the IGHV5-17/IGHJ4 pairing, while the MF59 group exhibited elevated frequencies of IGHV9-3/IGHJ3 and IGHV1-62/IGHJ2. Both the CpG 1018 and KA101 groups showed reduced IGHV4-1/IGHJ3 pairing frequencies, with the latter also displaying an increased IGHV1-72/IGHJ2 frequency (**Supplementary Figure 4**). Clonal diversity (Shannon index) and clonal concentration (D50) analyses showed that the Poly(I:C) group had the lowest values for both metrics, reflecting markedly reduced clonal diversity and highly concentrated clonal frequencies, consistent with robust oligoclonal expansion. In contrast, the KA101 and MF59 groups exhibited the highest values for both indices, indicating the most diverse B cell repertoires with dispersed clonal distributions and no evident dominant clones. The aluminum adjuvant, LNP, and CpG 1018 groups displayed clonal structures intermediate between these extremes.

Collectively, these findings demonstrate that distinct adjuvant formulations establish unique B-cell immune repertoire architectures by differentially modulating V gene usage preferences, V-J pairing selection, and clonal expansion patterns. MF59 and KA101 preferentially induce broad, highly diverse polyclonal humoral responses, whereas Poly(I:C) promotes oligoclonal restriction. These observations identify adjuvant composition as a critical determinant governing both the magnitude and breadth of B-cell immune responses.

## 4 Discussion

Cell-mediated immunity plays a critical role in controlling the reactivation of latent VZV infection and blocking viral transmission (35, 36).As the most abundant membrane protein on the VZV surface, gE has become the core antigen of VZV subunit vaccines, but its immunogenicity is highly dependent on effective adjuvant enhancement (37). In this study, we systematically compared the immune response characteristics of six mechanistically distinct adjuvants formulated with VZV gE antigen and elucidated their differential regulatory effects on innate, humoral, and cellular immunity via multi-omics analysis. These results deepen the understanding of adjuvant mechanisms and provide important experimental evidence for the rational design of VZV subunit vaccines.

Aluminum hydroxide induced weak innate immune activation, with detectable IL-1β responses but limited overall inflammation, ultimately resulting in an extreme Th2-biased response characterized by the lowest IgG2c/IgG1 ratio and absent IFN-γ^+^ T cell responses. These results align with previously reported immunological profiles of alum-adjuvanted gE vaccines (38). Notably, although aluminum adjuvants induced high levels of neutralizing antibodies, humoral immunity alone may be insufficient for long-term protection against this latent virus, as viral reactivation control primarily depends on cell-mediated immunity (39).

The squalene-in-water emulsion MF59 exhibited unique immunomodulatory characteristics, inducing moderate increases in IL-6, MCP-1, and IL-1β, along with the highest clonal diversity in the BCR immunorepertoire. This observation is consistent with the known role of squalene emulsions in promoting germinal center B cell differentiation and sustaining immune responses (40, 41). The Th1/Th2 mixed response induced by MF59 suggests that oil-in-water emulsions may provide broader antigen exposure for B cell responses through antigen depot formation and local inflammatory microenvironment remodeling (42). However, despite high neutralizing antibody titers, MF59 did not significantly outperform aluminum adjuvants, indicating its advantage may lie in immune response breadth and memory persistence rather than peak antibody levels.

CpG 1018 (a TLR9 agonist) exhibited strong Th1-polarizing characteristics, inducing the highest levels of IFN-α, IL-12p70, and IP-10, along with significant CD8^+^ T cell expansion and IFN-γ^+^, TNF-α^+^, and IL-2^+^ T cell responses, albeit with relatively lower neutralizing antibody levels. Mechanistically, CpG 1018 extensively enriched T cell receptor signaling pathways, cytokine-cytokine receptor interactions, and NF-κB signaling pathways, driving lymphocyte proliferation and Th1 polarization but lacking B cell activation co-stimulatory signals. This aligns with other studies showing that while TLR9 agonists alone induce antibody responses, coordinated, robust humoral and cellular immunity requires combination with aluminum adjuvants (38).

Poly(I:C), a TLR3 agonist, induced type I interferon responses and an antiviral state, with a profile similar to CpG 1018 but considerably lower magnitude. This may stem from differences in expression patterns of TLR3 versus TLR9 among APC subsets, distinct intracellular localization, and differential downstream signal transduction. In addition, Poly(I:C) induced more pronounced oligoclonal expansion (reflected by a markedly reduced D50 index), suggesting that while it activates strong antigen-specific B cell responses, limited clonal diversity may compromise the breadth of protection against viral variants.

In this study, LNPs exhibited innate immune activation characterized by MCP-1-driven monocyte and macrophage recruitment and significant enhancement of CD40L^+^CD4^+^ T cell responses. Transcriptome analysis showed that LNPs were involved in both TLR/MyD88 and RIG-I signaling, upregulating co-stimulatory molecules and homing receptors, and preferentially driving germinal center reactions, plasma cell differentiation, and memory cell establishment. This finding is consistent with the mechanism by which LNPs in mRNA vaccines activate TLR4 and STING pathways through ionizable lipids (43, 44). However, while LNP-induced neutralizing antibody titers exceeded those of TLR agonists alone, they did not reach KA101 levels, suggesting overly broad, imprecise inflammatory activation. Thus, while LNPs excel as nucleic acid delivery systems, their utility as protein subunit vaccine adjuvants requires refinement for more precise immunomodulation.

KA101 is a liposomal adjuvant with the same qualitative formulation as AS01 (DOPC, cholesterol, MPL, QS-21), but differs in its MPL source: KA101 uses a fully synthetic MPL that offers higher purity and a more defined molecular composition, whereas AS01 uses MPL naturally derived from *Salmonella minnesota*. The AS01 adjuvant system—which shares the same immunostimulants (MPL and QS-21) as AS01E—has been clinically demonstrated to elicit robust CD4^+^ T-cell and antibody responses (45). Systems biology studies have confirmed that the TLR4 agonist MPL and NLRP3 activator QS-21 in AS01 generate complex synergistic, additive, and emergent effects—unique gene expression patterns uninducible by either component alone (46). In this study, KA101 rapidly induced early IFN-γ production, consistent with AS01 triggering NK and CD8^+^ T cell IFN-γ secretion within 6 hours in draining lymph nodes (46). Of note, although IL-12p70 was undetectable at 20 h post-immunization, this does not contradict the robust IFN-γ response. Given that the kinetics of vaccine-induced IL-12p70 are highly dependent on the adjuvant formulation, our 20 h sampling time point likely fell within its declining phase; the precise profile of KA101-induced IL-12p70 awaits time-course characterization. Furthermore, IFN-γ can also be induced via IL-12p70-independent pathways, such as type I interferons directly activating NK and T cells, which may contribute to the high IFN-γ levels observed (47, 48). Collectively, these findings are further substantiated by splenic transcriptomic analysis, which revealed significant enrichment of T–B cell interaction signals and antigen presentation-related pathways, alongside markedly increased proportions of IFN-γ^+^ and IL-2^+^CD4^+^ and CD8^+^ T cells. Importantly, KA101 maintained high diverse B cell repertoire (Shannon and D50 indices comparable to MF59) while driving balanced Th1/Th2 responses and high neutralizing antibody levels, indicating efficient Tfh-B cell collaboration achieves optimal cellular-humoral immunity balance. This is highly consistent with the at least 11-year protective efficacy of the Shingrix vaccine shown in clinical trials (17, 49) and the key role of IL-2^+^ memory T cells as a correlate of protection (50).

Several limitations of this study should be noted. First, all experiments were performed in a mouse model, and although the adjuvants evaluated here (e.g., CpG 1018, MF59, and KA101) have well-characterized immunological mechanisms across species, caution should be exercised when extrapolating these findings to humans. Second, our transcriptomic and cellular analyses were performed on splenocytes rather than draining lymph nodes, the primary sites of antigen presentation and T cell priming. Third, bulk RNA-seq analysis of whole spleen tissue does not resolve the cellular heterogeneity underlying the observed transcriptional differences among adjuvant groups. Given that vaccine development against latent viruses such as VZV necessitates durable cell-mediated immunity to control viral reactivation; while our findings suggest that AS01-class complex adjuvants represent a promising strategy through early IFN-γ responses that coordinate Th1/Th2 polarization with germinal center reactions, these observations require validation in aged populations and latent infection models. Addressing these limitations, future studies employing single-cell RNA sequencing, multi-parameter flow cytometry, or cell sorting-based transcriptomic profiling, as well as analyses of draining lymph nodes in non-human primates or clinical samples, would be valuable to delineate adjuvant-specific effects on T cell subset composition and differentiation, validate the translational potential of our findings, and further investigate dose optimization and synergistic mechanisms among composite adjuvant components.

## 5 Conclusions

This study systematically compared the characteristics and mechanisms of immune response regulation among six different types of adjuvants formulated with the VZV gE antigen in a mouse model. The comparison was performed across multiple dimensions, including innate immunity, humoral immunity, cellular immunity, transcriptomics, and the BCR immune repertoire. Traditional adjuvants Al(OH)_3_ and MF59 induced weak innate immune responses and limited cellular immunity, despite eliciting high titers of neutralizing antibodies. Poly(I:C) displayed a Th2-skewed profile with partial Th1 tendencies and exhibited oligoclonal B-cell expansion, whereas CpG 1018 potently induced Th1-polarized responses. In contrast, the novel adjuvant LNP and the composite adjuvant KA101 activated comprehensive immune responses. Notably, KA101 induced robust innate immunity, high levels of neutralizing antibodies, while eliciting balanced Th1/Th2. Transcriptomic analysis further revealed that KA101 promoted germinal center reactions and long-term immune memory formation, along with the highest B cell repertoire diversity, demonstrating synergistic potential to induce cellular immunity, humoral immunity, and durable memory. Collectively, this study elucidated the immunomodulatory profiles and application advantages of six adjuvants, providing crucial preclinical evidence for adjuvant selection and formulation optimization of next-generation subunit herpes zoster vaccines.

## Data Availability

The data that support the findings of this study are available from the corresponding author upon reasonable request. Raw sequence data were uploaded to CNCB-NGDC (Accession: CRA041084 & CRA041101).
