# Supplementary material for: KA101 outperforms other clinical adjuvants in inducing balanced Th1/Th2 immunity and robust B cell responses to varicella-zoster virus glycoprotein E
Source: Front Immunol. 2026 Jul 16;17:1849099. doi: 10.3389/fimmu.2026.1849099 (PMC13422451; doi:10.3389/fimmu.2026.1849099)
Supplement: Supplementary file 1 [file DataSheet1.doc]

**Supplementary Materials**

Raw sequence data were uploaded to CNCB-NGDC (Accession: CRA041084). Shared URL:

https://ngdc.cncb.ac.cn/gsa/s/216JEK9V


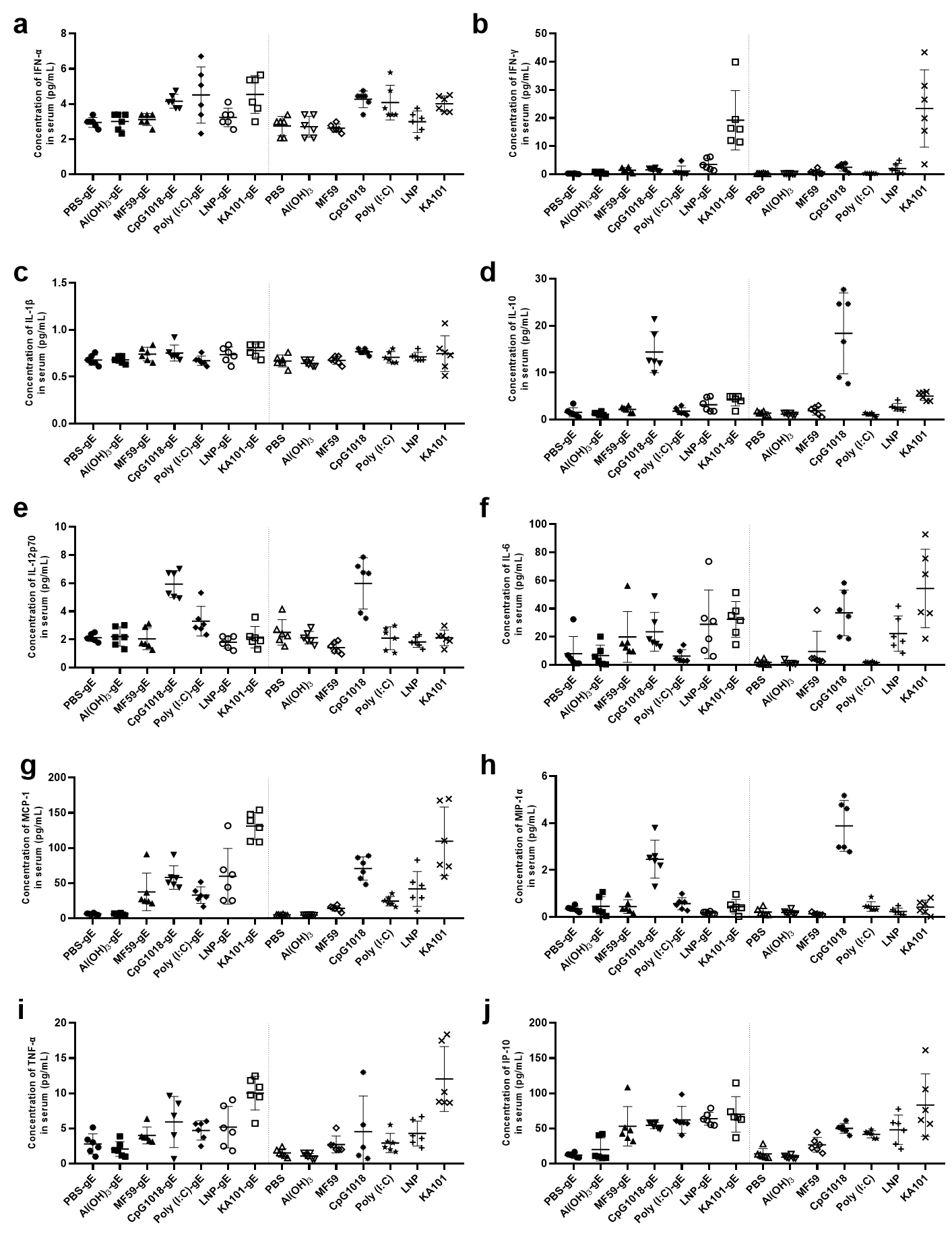


Figure S1. Concentrations of the indicated innate cytokines and chemokines were quantified by Luminex assay. Serum was collected from mice 20 hours after primary immunization.


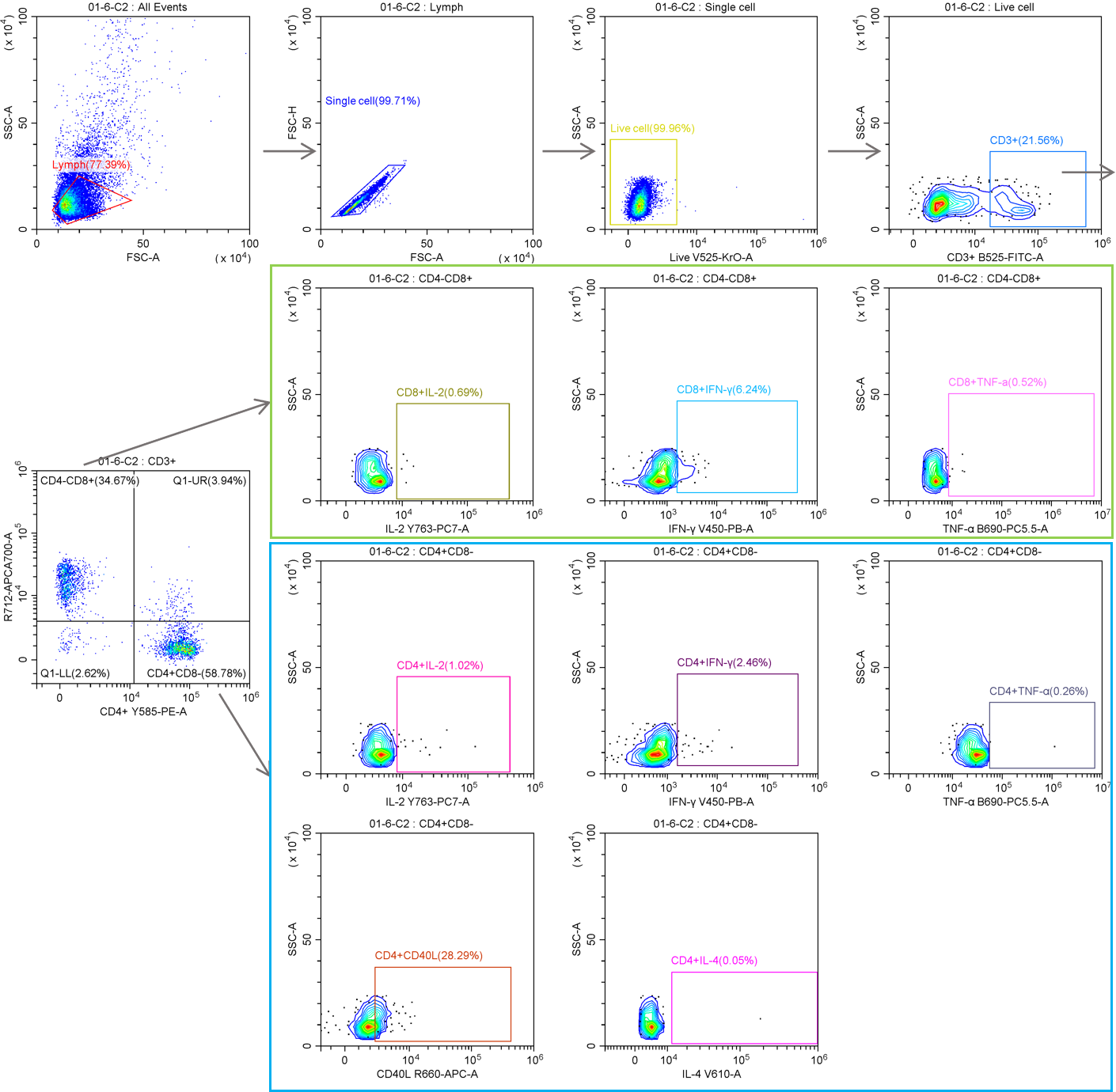


Figure S2. Gating strategy for identifying antigen-specific cytokine-producing T cells.

Splenocytes from immunized mice were stimulated with the gE peptide pool for 12 hours, followed by sequential gating on live, singlet lymphocytes, identification of CD4⁺ and CD8⁺ T cells, and analysis of intracellular cytokine expression.


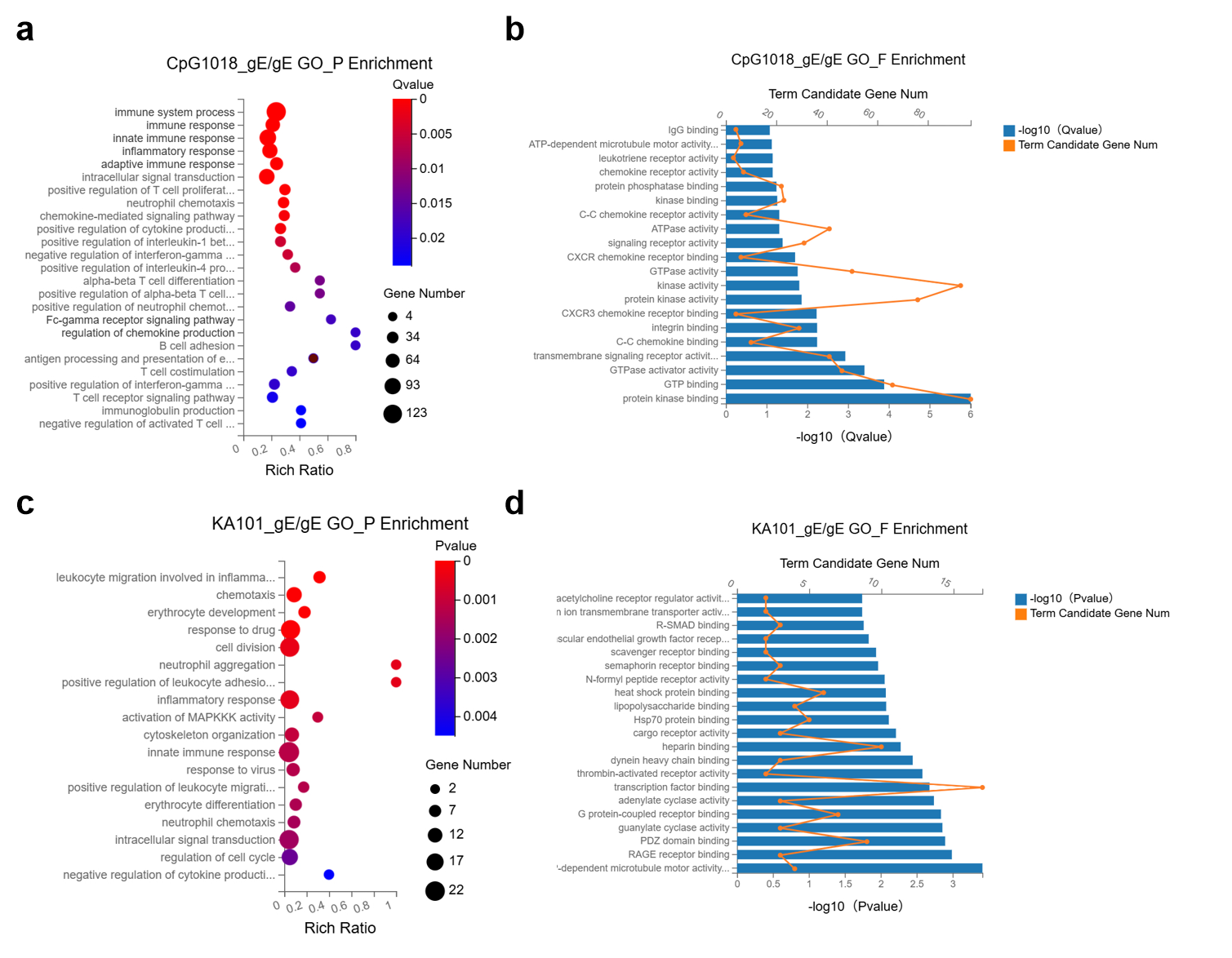


Figure S3. Enrichment analysis of GO terms. The most significantly enriched GO terms are shown for comparisons between (a,b) CpG 1018_gE and gE alone, and (c,d) KA101_gE and gE alone. Specifically, (a) and (c) display GO_P (Gene Ontology Biological Process) terms, and (b) and (d) display GO_F (Gene Ontology Molecular Function) terms.

| Table S1. Detailed KEGG pathway and GO enrichment analyses of the gE alone group versus the PBS control group. | | | | |
| --- | --- | --- | --- | --- |
| **KEGG Pathway Term ID** | **KEGG Pathway Term Desc** | **Rich Ratio** | **P value** | **Q value** |
| 04010 | MAPK signaling pathway | 0.0136 | 0.0000 | 0.0012 |
| 04612 | Antigen processing and presentation | 0.0241 | 0.0013 | 0.0113 |
| 04657 | IL-17 signaling pathway | 0.0220 | 0.0016 | 0.0121 |
| 04668 | TNF signaling pathway | 0.0177 | 0.0024 | 0.0152 |
| **GO_P Term ID** | **GO_P Term Desc** | **Rich Ratio** | **P value** | **Q value** |
| GO:0046632 | alpha-beta T cell differentiation | 0.0909 | 0.0143 | 0.0362 |
| GO:0045830 | positive regulation of isotype switching | 0.0769 | 0.0169 | 0.0393 |

| Table S2. Detailed KEGG pathway and GO enrichment analyses of the Al(OH)3_gE group versus the gE alone group. | | | | |
| --- | --- | --- | --- | --- |
| **KEGG Pathway Term ID** | **KEGG Pathway Term Desc** | **Rich Ratio** | **P value** | **Q value** |
| 04010 | MAPK signaling pathway | 0.0136 | 0.0001 | 0.0059 |
| 04612 | Antigen processing and presentation | 0.0241 | 0.0031 | 0.0321 |
| 04657 | IL-17 signaling pathway | 0.0220 | 0.0037 | 0.0321 |
| 04640 | Hematopoietic cell lineage | 0.0217 | 0.0037 | 0.0321 |
| 04625 | C-type lectin receptor signaling pathway | 0.0179 | 0.0055 | 0.0363 |
| **GO_F Term ID** | **GO_F Term Desc** | **Rich Ratio** | **P value** | **Q value** |
| GO:0045504 | dynein heavy chain binding | 0.1333 | 0.0002 | 0.0088 |
| GO:0051434 | BH3 domain binding | 0.1111 | 0.0132 | 0.0714 |
| GO:0030165 | PDZ domain binding | 0.0163 | 0.0141 | 0.0714 |
| GO:0008135 | translation factor activity, RNA binding | 0.0833 | 0.0175 | 0.0747 |
| GO:0000900 | translation repressor activity, mRNA regulatory element binding | 0.0769 | 0.0190 | 0.0747 |
| GO:0045503 | dynein light chain binding | 0.0769 | 0.0190 | 0.0747 |
| GO:0045182 | translation regulator activity | 0.0417 | 0.0348 | 0.1060 |
| GO:0035925 | mRNA 3'-UTR AU-rich region binding | 0.0370 | 0.0390 | 0.1161 |
| GO:0000146 | microfilament motor activity | 0.0333 | 0.0433 | 0.1228 |
| **GO_P Term ID** | **GO_P Term Desc** | **Rich Ratio** | **P value** | **Q value** |
| GO:0035710 | CD4-positive, alpha-beta T cell activation | 0.3333 | 0.0044 | 0.0393 |
| GO:0002408 | myeloid dendritic cell chemotaxis | 0.2500 | 0.0058 | 0.0393 |
| GO:0043367 | CD4-positive, alpha-beta T cell differentiation | 0.1667 | 0.0087 | 0.0467 |
| GO:2000553 | positive regulation of T-helper 2 cell cytokine production | 0.1111 | 0.0130 | 0.0485 |
| GO:0050778 | positive regulation of immune response | 0.0909 | 0.0159 | 0.0557 |
| GO:0045087 | innate immune response | 0.0059 | 0.0369 | 0.0786 |
| GO:0051607 | defense response to virus | 0.0090 | 0.0412 | 0.0828 |

| Table S3. Detailed KEGG pathway and GO enrichment analyses of the MF59_gE group versus the gE alone group. | | | | |
| --- | --- | --- | --- | --- |
| **KEGG Pathway Term ID** | **KEGG Pathway Term Desc** | **Rich Ratio** | **P value** | **Q value** |
| 04512 | ECM-receptor interaction | 0.0568 | 0.0001 | 0.0082 |
| 04510 | Focal adhesion | 0.0249 | 0.0046 | 0.1410 |
| 05310 | Asthma | 0.0833 | 0.0075 | 0.1410 |
| 04610 | Complement and coagulation cascades | 0.0326 | 0.0138 | 0.1878 |
| 04620 | Toll-like receptor signaling pathway | 0.0303 | 0.0167 | 0.1878 |
| 04612 | Antigen processing and presentation | 0.0361 | 0.0104 | 0.1724 |
| **GO_F Term ID** | **GO_F Term Desc** | **Rich Ratio** | **P value** | **Q value** |
| GO:0030020 | extracellular matrix structural constituent conferring tensile strength | 0.1282 | 0.0000 | 0.0001 |
| GO:0005539 | glycosaminoglycan binding | 0.1481 | 0.0000 | 0.0006 |
| GO:0008201 | heparin binding | 0.0389 | 0.0000 | 0.0009 |
| GO:0005201 | extracellular matrix structural constituent | 0.0407 | 0.0002 | 0.0081 |
| GO:0030021 | extracellular matrix structural constituent conferring compression resistance | 0.1538 | 0.0014 | 0.0377 |
| GO:0038085 | vascular endothelial growth factor binding | 0.2000 | 0.0212 | 0.1063 |
| GO:1904399 | heparan sulfate binding | 0.2000 | 0.0212 | 0.1063 |
| GO:0004896 | cytokine receptor activity | 0.0351 | 0.0247 | 0.1099 |
| GO:0008083 | growth factor activity | 0.0195 | 0.0283 | 0.1106 |
| GO:0070891 | lipoteichoic acid binding | 0.1429 | 0.0295 | 0.1106 |
| GO:0001851 | complement component C3b binding | 0.1250 | 0.0337 | 0.1190 |
| GO:0031072 | heat shock protein binding | 0.0241 | 0.0491 | 0.1419 |
| **GO_P Term ID** | **GO_P Term Desc** | **Rich Ratio** | **P value** | **Q value** |
| GO:0001780 | neutrophil homeostasis | 0.2727 | 0.0000 | 0.0047 |
| GO:0045087 | innate immune response | 0.0197 | 0.0001 | 0.0118 |
| GO:0002232 | leukocyte chemotaxis involved in inflammatory response | 0.2500 | 0.0005 | 0.0253 |
| GO:0019731 | antibacterial humoral response | 0.0460 | 0.0005 | 0.0257 |
| GO:0007155 | cell adhesion | 0.0146 | 0.0014 | 0.0474 |
| GO:0033089 | positive regulation of T cell differentiation in thymus | 0.1538 | 0.0014 | 0.0474 |
| GO:0016064 | immunoglobulin mediated immune response | 0.1000 | 0.0033 | 0.0523 |
| GO:0001905 | activation of membrane attack complex | 1.0000 | 0.0043 | 0.0523 |
| GO:0032652 | regulation of interleukin-1 production | 1.0000 | 0.0043 | 0.0523 |
| GO:0072677 | eosinophil migration | 1.0000 | 0.0043 | 0.0523 |
| GO:0006954 | inflammatory response | 0.0158 | 0.0060 | 0.0660 |
| GO:0032753 | positive regulation of interleukin-4 production | 0.0741 | 0.0060 | 0.0660 |
| GO:0002376 | immune system process | 0.0134 | 0.0075 | 0.0660 |
| GO:1990959 | eosinophil homeostasis | 0.5000 | 0.0086 | 0.0660 |
| GO:0030198 | extracellular matrix organization | 0.0215 | 0.0086 | 0.0660 |
| GO:0034612 | response to tumor necrosis factor | 0.0556 | 0.0106 | 0.0690 |
| GO:0002434 | immune complex clearance | 0.3333 | 0.0129 | 0.0690 |
| GO:0002408 | myeloid dendritic cell chemotaxis | 0.2500 | 0.0172 | 0.0788 |
| GO:0071621 | granulocyte chemotaxis | 0.2500 | 0.0172 | 0.0788 |
| GO:0032642 | regulation of chemokine production | 0.2000 | 0.0214 | 0.0848 |
| GO:0002685 | regulation of leukocyte migration | 0.1667 | 0.0256 | 0.0901 |
| GO:0045629 | negative regulation of T-helper 2 cell differentiation | 0.1667 | 0.0256 | 0.0901 |
| GO:0001525 | angiogenesis | 0.0145 | 0.0313 | 0.0953 |
| GO:0045627 | positive regulation of T-helper 1 cell differentiation | 0.1250 | 0.0340 | 0.0969 |
| GO:1903053 | regulation of extracellular matrix organization | 0.1250 | 0.0340 | 0.0969 |
| GO:0001867 | complement activation, lectin pathway | 0.1111 | 0.0382 | 0.0993 |
| GO:0030449 | regulation of complement activation | 0.1111 | 0.0382 | 0.0993 |
| GO:0050671 | positive regulation of lymphocyte proliferation | 0.1111 | 0.0382 | 0.0993 |
| GO:0046632 | alpha-beta T cell differentiation | 0.0909 | 0.0465 | 0.1109 |
| GO:0050777 | negative regulation of immune response | 0.0909 | 0.0465 | 0.1109 |
| GO:0050778 | positive regulation of immune response | 0.0909 | 0.0465 | 0.1109 |
| GO:0034097 | response to cytokine | 0.0330 | 0.0072 | 0.0660 |

| Table S4. Detailed KEGG pathway and GO enrichment analyses of the Poly(I:C)_gE group versus the gE alone group. | | | | |
| --- | --- | --- | --- | --- |
| **KEGG Pathway Term ID** | **KEGG Pathway Term Desc** | **Rich Ratio** | **P value** | **Q value** |
| 05162 | Measles | 0.0280 | 0.0001 | 0.0145 |
| 04612 | Antigen processing and presentation | 0.0361 | 0.0005 | 0.0234 |
| 05145 | Toxoplasmosis | 0.0275 | 0.0011 | 0.0297 |
| 04010 | MAPK signaling pathway | 0.0136 | 0.0021 | 0.0316 |
| 05134 | Legionellosis | 0.0333 | 0.0059 | 0.0736 |
| 05170 | Human immunodeficiency virus 1 infection | 0.0128 | 0.0097 | 0.0874 |
| 05166 | Human T-cell leukemia virus 1 infection | 0.0123 | 0.0107 | 0.0874 |
| 05171 | Coronavirus disease - COVID-19 | 0.0123 | 0.0107 | 0.0874 |
| 04657 | IL-17 signaling pathway | 0.0220 | 0.0131 | 0.0968 |
| 04144 | Endocytosis | 0.0113 | 0.0137 | 0.0968 |
| 04668 | TNF signaling pathway | 0.0177 | 0.0197 | 0.1307 |
| **GO_F Term ID** | **GO_F Term Desc** | **Rich Ratio** | **P value** | **Q value** |
| GO:0048019 | receptor antagonist activity | 0.0909 | 0.0134 | 0.0492 |
| GO:0097677 | STAT family protein binding | 0.0769 | 0.0158 | 0.0496 |
| GO:0005545 | 1-phosphatidylinositol binding | 0.0714 | 0.0170 | 0.0496 |
| GO:0008201 | heparin binding | 0.0111 | 0.0204 | 0.0561 |
| GO:0043027 | cysteine-type endopeptidase inhibitor activity involved in apoptotic process | 0.0294 | 0.0409 | 0.0874 |
| GO:0044877 | protein-containing complex binding | 0.0045 | 0.0478 | 0.0927 |
| GO:0031072 | heat shock protein binding | 0.0241 | 0.0046 | 0.0288 |
| GO:0031625 | ubiquitin protein ligase binding | 0.0094 | 0.0068 | 0.0367 |
| GO:0003700 | DNA-binding transcription factor activity | 0.0062 | 0.0074 | 0.0367 |
| GO:0035497 | cAMP response element binding | 0.0556 | 0.0219 | 0.0585 |
| GO:0044389 | ubiquitin-like protein ligase binding | 0.0400 | 0.0302 | 0.0730 |
| GO:0051059 | NF-kappaB binding | 0.0286 | 0.0421 | 0.0874 |
| GO:0033613 | activating transcription factor binding | 0.0278 | 0.0433 | 0.0874 |
| **GO_P Term ID** | **GO_P Term Desc** | **Rich Ratio** | **P value** | **Q value** |
| GO:0019050 | suppression by virus of host apoptotic process | 0.5000 | 0.0025 | 0.0197 |
| GO:0051169 | nuclear transport | 0.2500 | 0.0050 | 0.0275 |
| GO:0034144 | negative regulation of toll-like receptor 4 signaling pathway | 0.1111 | 0.0113 | 0.0366 |
| GO:2001171 | positive regulation of ATP biosynthetic process | 0.0556 | 0.0225 | 0.0534 |
| GO:0005978 | glycogen biosynthetic process | 0.0526 | 0.0237 | 0.0545 |
| GO:0042981 | regulation of apoptotic process | 0.0098 | 0.0271 | 0.0561 |
| GO:0051607 | defense response to virus | 0.0090 | 0.0317 | 0.0628 |
| GO:0001678 | cellular glucose homeostasis | 0.0323 | 0.0384 | 0.0709 |
| GO:0010498 | proteasomal protein catabolic process | 0.0278 | 0.0444 | 0.0750 |
| GO:0060548 | negative regulation of cell death | 0.0190 | 0.0077 | 0.0336 |
| GO:0045657 | positive regulation of monocyte differentiation | 0.1000 | 0.0125 | 0.0383 |
| GO:0045830 | positive regulation of isotype switching | 0.0769 | 0.0163 | 0.0446 |
| GO:0045190 | isotype switching | 0.0526 | 0.0237 | 0.0545 |
| GO:0043032 | positive regulation of macrophage activation | 0.0476 | 0.0262 | 0.0557 |
| GO:0032496 | response to lipopolysaccharide | 0.0100 | 0.0264 | 0.0557 |
| GO:0030224 | monocyte differentiation | 0.0455 | 0.0274 | 0.0561 |
| GO:0001774 | microglial cell activation | 0.0345 | 0.0360 | 0.0693 |
| GO:0016192 | vesicle-mediated transport | 0.0075 | 0.0440 | 0.0750 |

| Table S5. Detailed KEGG pathway and GO enrichment analyses of the LNP_gE group versus the gE alone group. | | | | |
| --- | --- | --- | --- | --- |
| **KEGG Pathway Term ID** | **KEGG Pathway Term Desc** | **Rich Ratio** | **P value** | **Q value** |
| 04650 | Natural killer cell mediated cytotoxicity | 0.0268 | 0.0058 | 0.0703 |
| 04666 | Fc gamma R-mediated phagocytosis | 0.0217 | 0.0368 | 0.1649 |
| 04010 | MAPK signaling pathway | 0.0204 | 0.0003 | 0.0121 |
| 05162 | Measles | 0.0280 | 0.0012 | 0.0284 |
| 04612 | Antigen processing and presentation | 0.0361 | 0.0025 | 0.0449 |
| 04657 | IL-17 signaling pathway | 0.0330 | 0.0033 | 0.0471 |
| 05161 | Hepatitis B | 0.0184 | 0.0162 | 0.1320 |
| 04662 | B cell receptor signaling pathway | 0.0260 | 0.0265 | 0.1611 |
| 04658 | Th1 and Th2 cell differentiation | 0.0230 | 0.0332 | 0.1611 |
| 05235 | PD-L1 expression and PD-1 checkpoint pathway in cancer | 0.0227 | 0.0339 | 0.1611 |
| 04620 | Toll-like receptor signaling pathway | 0.0202 | 0.0421 | 0.1666 |
| 04660 | T cell receptor signaling pathway | 0.0194 | 0.0452 | 0.1666 |
| 04659 | Th17 cell differentiation | 0.0192 | 0.0460 | 0.1666 |
| **GO_F Term ID** | **GO_F Term Desc** | **Rich Ratio** | **P value** | **Q value** |
| GO:0038164 | thrombopoietin receptor activity | 1.0000 | 0.0021 | 0.0408 |
| GO:0005044 | scavenger receptor activity | 0.0417 | 0.0046 | 0.0497 |
| GO:0050839 | cell adhesion molecule binding | 0.0250 | 0.0124 | 0.0840 |
| GO:0031726 | CCR1 chemokine receptor binding | 0.1429 | 0.0147 | 0.0857 |
| GO:0031727 | CCR2 chemokine receptor binding | 0.1111 | 0.0188 | 0.0919 |
| GO:0048019 | receptor antagonist activity | 0.0909 | 0.0230 | 0.0919 |
| GO:0004697 | protein kinase C activity | 0.0625 | 0.0333 | 0.0969 |
| GO:0004698 | calcium-dependent protein kinase C activity | 0.0625 | 0.0333 | 0.0969 |
| GO:0071933 | Arp2/3 complex binding | 0.0588 | 0.0353 | 0.0969 |
| GO:0031434 | mitogen-activated protein kinase kinase binding | 0.0455 | 0.0454 | 0.1102 |
| GO:0017017 | MAP kinase tyrosine/serine/threonine phosphatase activity | 0.0769 | 0.0271 | 0.0969 |
| **GO_P Term ID** | **GO_P Term Desc** | **Rich Ratio** | **P value** | **Q value** |
| GO:0002215 | defense response to nematode | 0.5000 | 0.0000 | 0.0052 |
| GO:0030220 | platelet formation | 0.1053 | 0.0007 | 0.0258 |
| GO:0032693 | negative regulation of interleukin-10 production | 0.0870 | 0.0010 | 0.0258 |
| GO:0032753 | positive regulation of interleukin-4 production | 0.0741 | 0.0014 | 0.0258 |
| GO:0050728 | negative regulation of inflammatory response | 0.0270 | 0.0015 | 0.0258 |
| GO:0072677 | eosinophil migration | 1.0000 | 0.0020 | 0.0258 |
| GO:1990960 | basophil homeostasis | 1.0000 | 0.0020 | 0.0258 |
| GO:0006935 | chemotaxis | 0.0222 | 0.0026 | 0.0313 |
| GO:1990959 | eosinophil homeostasis | 0.5000 | 0.0041 | 0.0373 |
| GO:0043124 | negative regulation of I-kappaB kinase/NF-kappaB signaling | 0.0408 | 0.0045 | 0.0403 |
| GO:0032715 | negative regulation of interleukin-6 production | 0.0385 | 0.0050 | 0.0424 |
| GO:0035702 | monocyte homeostasis | 0.3333 | 0.0061 | 0.0424 |
| GO:0002408 | myeloid dendritic cell chemotaxis | 0.2500 | 0.0081 | 0.0461 |
| GO:0032642 | regulation of chemokine production | 0.2000 | 0.0101 | 0.0481 |
| GO:0071347 | cellular response to interleukin-1 | 0.0244 | 0.0121 | 0.0495 |
| GO:0043305 | negative regulation of mast cell degranulation | 0.1667 | 0.0122 | 0.0495 |
| GO:2000503 | positive regulation of natural killer cell chemotaxis | 0.1667 | 0.0122 | 0.0495 |
| GO:0032714 | negative regulation of interleukin-5 production | 0.1429 | 0.0142 | 0.0513 |
| GO:0150079 | negative regulation of neuroinflammatory response | 0.1429 | 0.0142 | 0.0513 |
| GO:0030219 | megakaryocyte differentiation | 0.1111 | 0.0182 | 0.0581 |
| GO:0034144 | negative regulation of toll-like receptor 4 signaling pathway | 0.1111 | 0.0182 | 0.0581 |
| GO:0050671 | positive regulation of lymphocyte proliferation | 0.1111 | 0.0182 | 0.0581 |
| GO:0045589 | regulation of regulatory T cell differentiation | 0.1000 | 0.0202 | 0.0609 |
| GO:0050764 | regulation of phagocytosis | 0.1000 | 0.0202 | 0.0609 |
| GO:0001780 | neutrophil homeostasis | 0.0909 | 0.0222 | 0.0632 |
| GO:0050777 | negative regulation of immune response | 0.0909 | 0.0222 | 0.0632 |
| GO:0006955 | immune response | 0.0100 | 0.0229 | 0.0632 |
| GO:0071356 | cellular response to tumor necrosis factor | 0.0154 | 0.0288 | 0.0702 |
| GO:0060216 | definitive hemopoiesis | 0.0556 | 0.0361 | 0.0773 |
| GO:0002526 | acute inflammatory response | 0.0500 | 0.0400 | 0.0809 |
| GO:0048245 | eosinophil chemotaxis | 0.0476 | 0.0419 | 0.0821 |
| GO:0090027 | negative regulation of monocyte chemotaxis | 0.1667 | 0.0122 | 0.0495 |
| GO:0034097 | response to cytokine | 0.0220 | 0.0148 | 0.0527 |
| GO:1903753 | negative regulation of p38MAPK cascade | 0.1250 | 0.0162 | 0.0549 |
| GO:0045657 | positive regulation of monocyte differentiation | 0.1000 | 0.0202 | 0.0609 |
| GO:1990869 | cellular response to chemokine | 0.0556 | 0.0361 | 0.0773 |
| GO:0030224 | monocyte differentiation | 0.0455 | 0.0439 | 0.0841 |


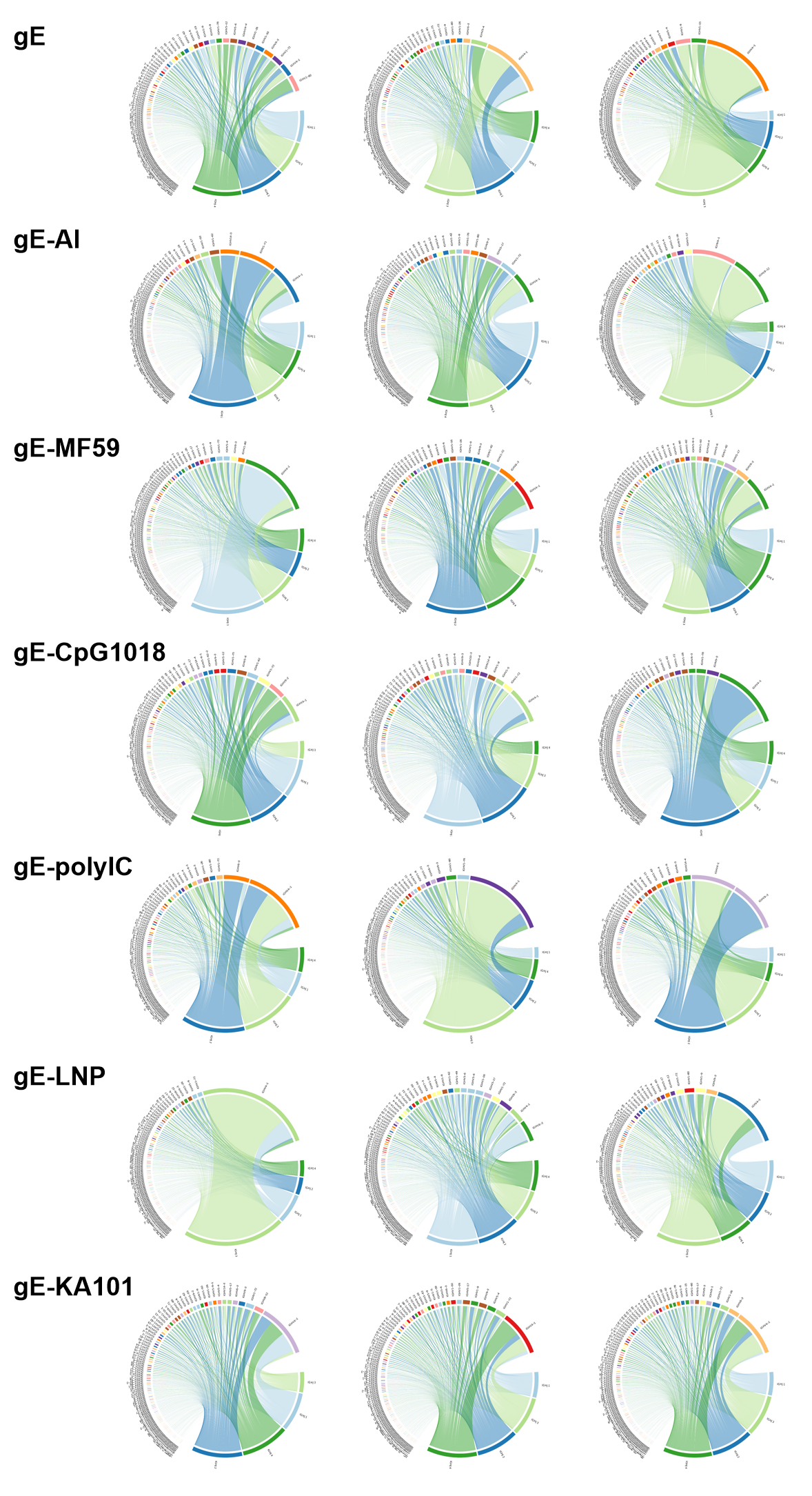


Figure S4. V-J gene pairing frequency distribution.
